# Neuromodulation of BAG co-chaperones by HIV-1 viral proteins and H_2_O_2_: implications for HIV-associated neurological disorders

**DOI:** 10.1038/s41420-021-00424-0

**Published:** 2021-03-26

**Authors:** Michael R. Duggan, Taha Mohseni Ahooyi, Vinay Parikh, Kamel Khalili

**Affiliations:** 1 Department of Neuroscience, Center for Neurovirology, Lewis Katz School of Medicine at Temple University, 3500 N. Broad Street, 7th Floor, Philadelphia, PA 19140 USA; 2 Department of Psychology, College of Liberal Arts at Temple University, 1701 N 13th Street, 9th Floor, Philadelphia, PA 19122 USA

**Keywords:** Cellular neuroscience, Molecular neuroscience

## Abstract

Despite increasing numbers of aged individuals living with HIV, the mechanisms underlying HIV-associated neurological disorders (HANDs) remain elusive. As HIV-1 pathogenesis and aging are characterized by oxidative stress as well as altered protein quality control (PQC), reactive oxygen species (ROS) themselves might constitute a molecular mediator of neuronal PQC by modulating BCL-2 associated athanogene (BAG) family members. Present results reveal H_2_O_2_ replicated and exacerbated a reduction in neuronal BAG3 induced by the expression of HIV-1 viral proteins (i.e., Tat and Nef), while also causing an upregulation of BAG1. Such a reciprocal regulation of BAG3 and BAG1 levels was also indicated in two animal models of HIV, the doxycycline-inducible Tat (iTat) and the Tg26 mouse. Inhibiting oxidative stress via antioxidants in primary culture was capable of partially preserving neuronal BAG3 levels as well as electrophysiological functioning otherwise altered by HIV-1 viral proteins. Current findings indicate HIV-1 viral proteins and H_2_O_2_ may mediate neuronal PQC by exerting synergistic effects on complementary BAG family members, and suggest novel therapeutic targets for the aging HIV-1 population.

## Introduction

Amongst persons living with HIV, the implementation of combined antiretroviral therapies (cARTs) since the mid-1990s has significantly increased life expectancies and decreased mortality rates such that approximately half of the HIV+ population is now ≥50 years of age^1^. However, this cohort is consistently found to be at a significantly greater risk for developing HIV-associated neurological disorders (HANDs), a spectrum of cognitive deficits reflecting impairments across several domains (e.g., executive functioning, memory, attention) and ensuing detriments on daily functioning^2–6^. Furthermore, despite the active debate surrounding the prevalence of HAND, it is evident HIV-infected individuals in the cART era illustrate significantly greater risk for the development and progression of neurocognitive deficits compared to the general population^7,8^. Thus, examining the neurobiological underpinnings of HAND appear warranted, including the potential consequences of HIV protein toxicity on homeostatic processes in neurons.

While its underlying mechanisms remain debated, evidence suggests the adverse effects of specific viral proteins may contribute to HAND, including the trans-activator of transcription (Tat) and the negative factor (Nef) proteins^9,10^. Tat can compromise a variety of homeostatic processes in the CNS, including neuronal viability, synapse formation, and neuroinflammatory profiles^11–13^. Furthermore, the consequences of Tat on cellular homeostasis may be due to its capacity to significantly increase levels of oxidative stress^14–17^. In addition, Nef may promote neuronal dysfunction through several complementary processes in the brain, including impaired metabolic activity, elevated neuronal cell death, and increased levels of neuroinflammation^18–21^. Consistent with the effect of Tat, elevations in oxidative stress are observed in response to Nef^22–24^. Moreover, these viral proteins may precipitate such increases in part by altering mitochondrial dynamics of neurons^25^.

Due to abnormal levels of reactive oxygen species (ROS), oxidative stress can induce variation in numerous homeostatic mechanisms that are associated with compromised neuronal functioning and neurodegenerative diseases^26^. H_2_O_2_ is particularly unique given its lack of an unpaired electron, which allows it to be more abundant and maintain the longest half-life compared to other ROS, in addition to its capacity to permeate lipid bilayers^27^. While increased measures of oxidative stress are commonly observed in the CNS of HIV-1 infected individuals, accumulating evidence suggests such elevations can compromise efficient neuronal functioning and may contribute to HAND^28,29^. Similarly, the aging CNS is correlated with elevations in oxidative stress, and such ROS imbalances are associated with impaired neuronal functioning that is otherwise observed in age-related neurodegenerative diseases, including Alzheimer’s disease^30–32^. Thus, oxidative stress, particularly H_2_O_2_, could mediate the deleterious effects induced by HIV-1 viral proteins in the aging brain.

Along with abnormal ROS, both HIV infection and aging are associated with alterations in neuronal protein quality control (PQC)^33–35^. Through their shared BAG domain, the BCL-2 associated athanogene (BAG) family of co-chaperones are necessary for efficient PQC by functioning as nucleotide exchange factors, thereby modulating the activity of Hsp70 via its ATPase domain^36,37^. BAG3, in addition to stimulating removal of malformed or aggregated polypeptides via autophagy, is emerging as a crucial regulator of stress granules (SGs), membrane-less compartments that can facilitate aberrant accumulation of aggregated proteins^38,39^. Along with its potent anti-apoptotic properties, growing data also suggest BAG1 levels are inversely proportional to BAG3, potentially due to its complementary clearance of substrates via the ubiquitin proteasomal system (UPS)^40–42^. Such a functional transition between BAG3 and BAG1 dependent PQC is postulated to be an adaptive preservation of neuronal homeostasis under conditions that might otherwise facilitate neuropathogenesis, such as viral infection, aging, and elevated oxidative stress^43,44^. Therefore, in response to HIV-1 viral proteins or aging, H_2_O_2_ itself could constitute an important modulator of neuronal PQC by regulating BAG family members.

Consistent with this hypothesis, recent reports suggest variation in certain BAG family members can be induced by HIV-1 Tat, as well as aging and ROS conditions^42,45,46^. Investigations from our laboratory have demonstrated that a significant downregulation of BAG3 is characteristic of HIV-transgenic mouse models as well as primary rat neurons expressing the Tat protein^46^. Furthermore, an inverse relationship between BAG3 and BAG1 levels has been reported in cell lines exposed to H_2_O_2_, in multiple brain regions of aged mice, as well as hippocampal slices from aged rats^42,45^. However, it remains unclear how elevations in H_2_O_2_ may directly modulate BAG family members in primary neurons, particularly in comparison to HIV-1 proteins.

To address this aim, the current experiments interrogate the effects of viral proteins Tat and Nef as well as H_2_O_2_ in primary neurons cultured from rat embryos. Findings reveal a reduction in neuronal BAG3 induced by the expression of HIV-1 viral proteins (i.e., Tat and Nef) was emulated and exacerbated by H_2_O_2_, which also caused an upregulation of BAG1. Inhibiting oxidative stress via antioxidants in primary culture was capable of partially preserving neuronal BAG3 levels as well as electrophysiological functioning otherwise altered by HIV-1 viral proteins. Therefore, the current study provides mechanistic insights relevant to treatment development for our aging HIV-1 population.

## Results

### HIV-1 viral proteins (Tat and Nef) decrease BAG3 and increase ROS

**Fig. 1:**
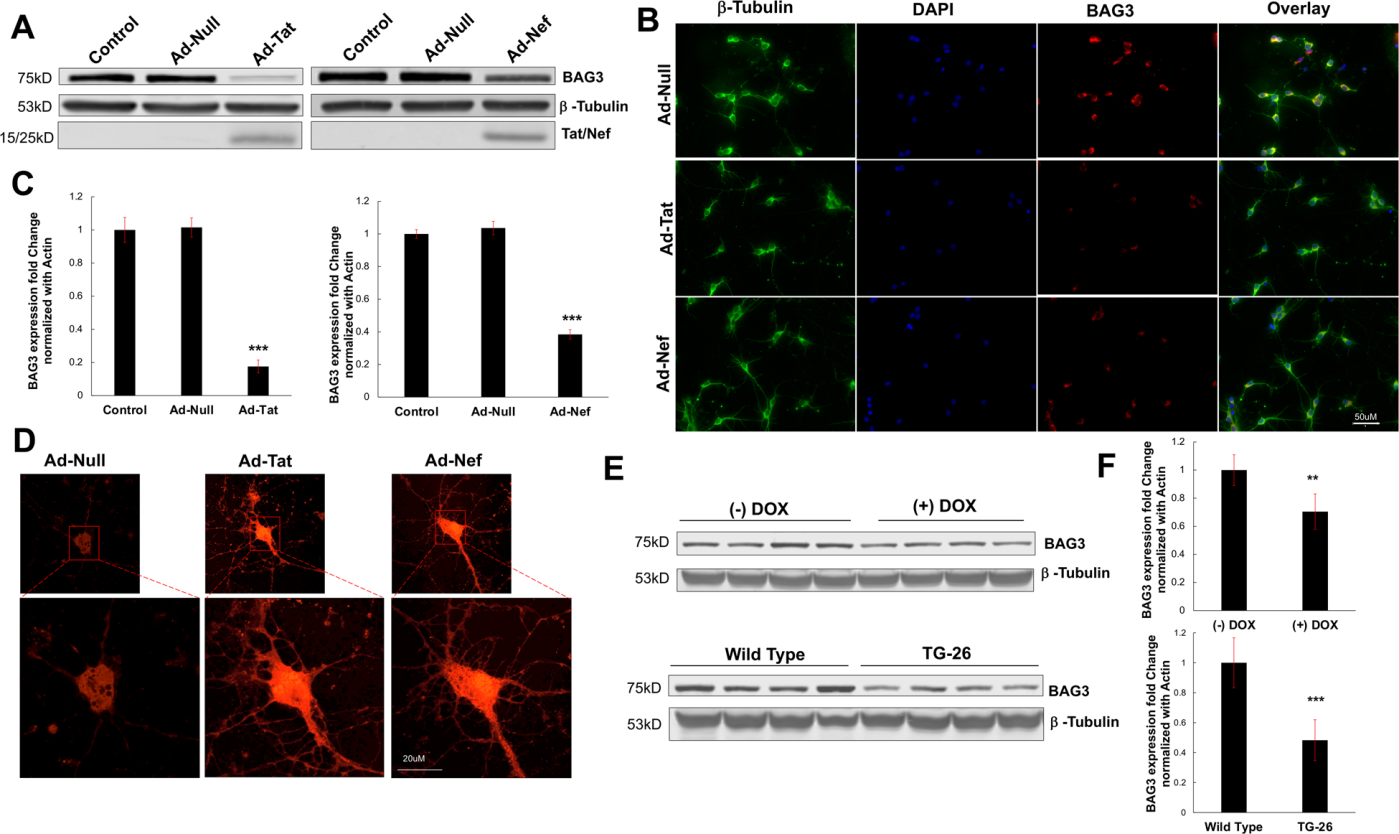
HIV-1 proteins (Tat and Nef) decrease BAG3 and increase ROS. **A** Western blot results illustrated the neuronal expression of either HIV-1 viral protein, Tat, or Nef, resulted in decreased protein levels of BAG3. **B** Immunocytochemistry analysis validated the reduction in BAG3 protein when compared to control conditions. **C** qPCR results further confirmed the effect of HIV-1 viral proteins on BAG3, demonstrating a significant decrease in its RNA following Tat or Nef expression. **D** Live-cell microscopy indicated significantly increased ROS in neurons exposed to either Tat or Nef. **E** Results from the frontal cortex of two HIV animal models indicated minor decreases in BAG3 protein were maintained in vivo, particularly in Tg26 mice. **F** qPCR results indicated significant in vivo reductions in BAG3 RNA across both HIV animal models. **p < 0.01; ***p < 0.001. Scale bar equals 50 μM; ×20 magnification. Scale bar equals 20 μM; ×60 magnification.

Primary neurons expressing HIV-1 Tat displayed significantly reduced levels of BAG3, consistent with previous observations from our laboratory^46^. Immunoblotting and fluorescent microscopy data demonstrated a significant decrease in BAG3 protein in response to Tat (Fig. 1A, B; Supplementary Fig. 1A, B). Similarly, we found the expression of HIV-1 Nef in primary neurons significantly attenuated BAG3 protein (Fig. 1A, B; Supplementary Fig. 1A, B). Moreover, both Tat and Nef were capable of independently downregulating BAG3 RNA (Fig. 1C). In assessing variations in oxidative stress due to such viral proteins, live-cell confocal microscopy illustrated significant elevations ROS following the expression of Tat or Nef (Fig. 1D; Supplementary Fig. 1C).

To determine if the pattern of BAG3 downregulation associated with viral proteins is maintained in vivo, cortical tissue from two distinct HIV animal models (iTat and Tg26) was analyzed. In a doxycycline-inducible mouse model (iTat), decreases in BAG3 protein and RNA were detected in response to Tat (Fig. 1E; Supplementary Fig. 1D). In Tg26-HIV mice, which express multiple viral proteins including Tat and Nef, more abundant significant reductions in BAG3 levels were observed (Fig. 1E, F; Supplementary Fig. 1D). Together, initial findings indicate the viral proteins Tat and Nef are capable of inducing oxidative stress in neurons, while concomitantly mitigating BAG3 levels.

### H_2_O_2_ treatment conditions

Next, we sought to examine if oxidative stress via H_2_O_2_ was capable of regulating neuronal BAG family members independent of HIV-1 viral protein expression. However, the optimal treatment parameters for H_2_O_2_ in primary neuronal culture are not well-established, due to the lack of studies employing H_2_O_2_ in primary neuronal cultures as well as the reliability of techniques used to assess its kinetics^47–50^. Based on the conditions and techniques available in the limited literature, several experiments were initially conducted to determine the parameters of H_2_O_2_ that alter general metabolic activity without compromising viability.

At a fixed duration of 6 h, H_2_O_2_ significantly decreased metabolic activity at dosages above 150 μM as indicated by MTT assay, while trypan blue staining suggested cell viability was significantly mitigated at concentrations above 250 μM (Supplementary Fig. 2A, B). Utilizing a 250 μM concentration, time-course experiments illustrated significantly decreased metabolism between 2 and 6 h of treatment, while significant increased cell death was observed after 8 h of treatment (Supplementary Fig. 2C, D). Therefore, such fixed parameters (i.e., 250 μM, 6 h) were selected as the optimal treatment conditions in succeeding experiments.

### H_2_O_2_ induces BAG3 reduction

**Fig. 2:**
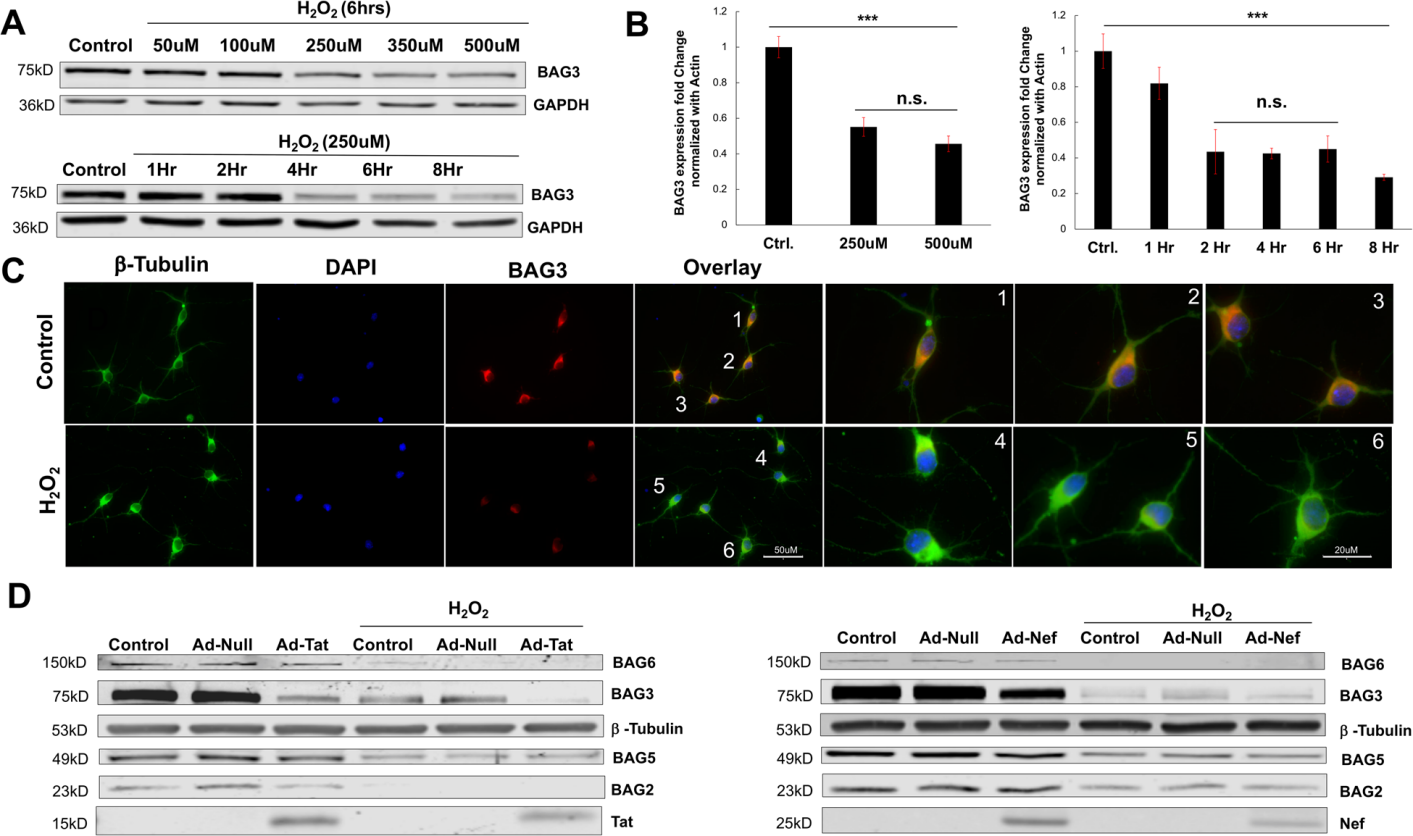
H_2_O_2_ induces BAG3 reduction. **A** Immunoblot results indicated H_2_O_2_ treatment caused time and dosage-dependent decreases in neuronal BAG3. **B** qPCR demonstrated H_2_O_2_ treatment causes significant decreases in neuronal BAG3 RNA. **C** In addition to validating H_2_O_2_-induced reductions in BAG3 protein, immunocytochemistry suggested neurons exposed to H_2_O_2_ treatment may develop minor dystrophies in their branching processes. **D** Immunoblotting revealed H_2_O_2_ reduced other BAG family members (BAG2, BAG5, BAG6), and exacerbated the reduction in BAG3 observed in response to Tat or Nef. ***p < 0.001. Scale bar equals 50 μM; ×40 magnification. Scale bar equals 20 μM; ×63 magnification.

In primary neurons exposed to H_2_O_2_, significant reductions in BAG3 were observed. Dosage experiments revealed concentrations of 250 μM and above significantly decreased BAG3 protein and RNA (Fig. 2A, B). While using this fixed dosage, time-course experiments illustrated such decreases in BAG3 protein were evident at approximately 4 h post-treatment, while significant reductions in RNA precede this reduction by ~2 h (Fig. 2A, B). Fluorescent microscopy validated such significant reductions in BAG3 following treatment of 250 μM for 6 h; furthermore, this illuminated morphological alterations in neurons exposed to H_2_O_2_, with some of these cells developing dystrophy in their processes compared to control conditions (Fig. 2C; Supplementary Fig. 3A). Interestingly, treatment of primary neurons with moderate dosages (100 μM) for extended durations (6–8 h) emulated these declines in BAG3 protein, while minimal dosages (50 μM) were unsuccessful in perturbing BAG3 protein levels at any time point (Supplementary Fig. 3B).

Strikingly, the combination of H_2_O_2_ and either viral protein Tat or Nef resulted in an additive effect, exacerbating the reduction in BAG3 (Fig. 2D). Such an additive effect was not observed when primary neurons co-expressed both viral proteins (Supplementary Fig. 3C). Moreover, decreases in BAG3 appeared to be neuronal-specific, provided that primary rat astrocytes failed to show similar sensitivity to H_2_O_2_ treatment (Supplementary Fig. 3D). In addition to BAG3, immunoblotting analysis also suggested H_2_O_2_ treatment reduced the protein levels of several other BAG family members, namely BAG6, BAG5, and BAG2 (Fig. 2D). Together, these data revealed the capacity of H_2_O_2_ to directly alter levels of BAG family members in both the transcription and translation levels, particularly BAG3, and support the postulation that oxidative stress is an important regulator of neuronal BAG proteins.

### Compensatory BAG1 regulation

**Fig. 3:**
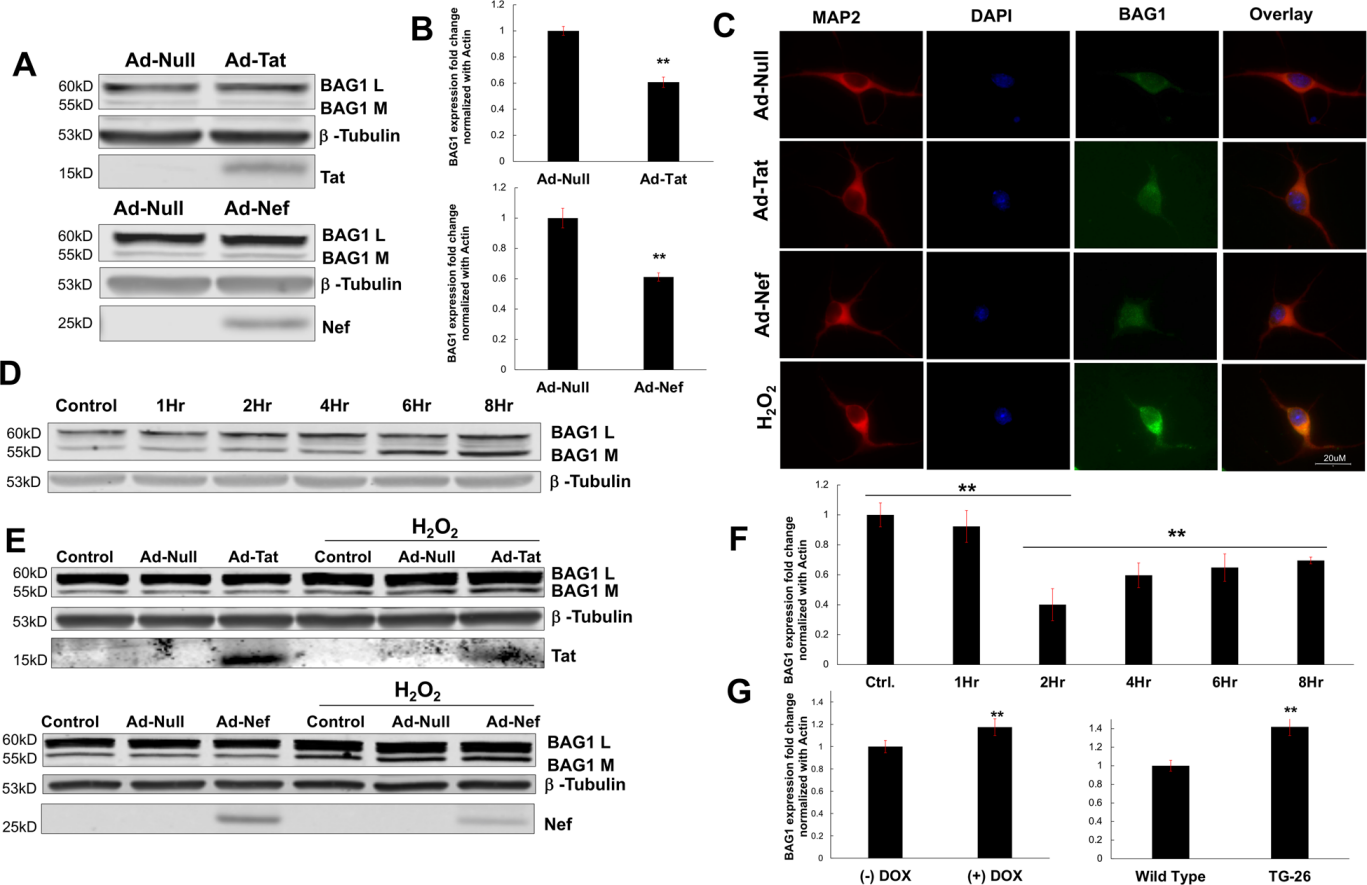
Compensatory BAG1 regulation. **A** Western blotting suggested neurons expressing either Tat or Nef did not display altered levels of BAG1 protein in either of its two predominant neuronal isoforms, L or M. **B** qPCR data suggested each viral protein downregulates levels of BAG1 RNA, despite a lack of alterations at the protein level. **C** Immunocytochemistry indicated H_2_O_2_ induces a compensatory increase in neuronal BAG1 protein. **D** Western blot results demonstrated such H_2_O_2_-induced increases in BAG1 were dependent on the duration of H_2_O_2_ treatment. **E** Immunoblot analyses illustrated H_2_O_2_ treatment in the context of Tat or Nef did not exacerbate the elevation in BAG1 protein. **F** qPCR data revealed an initial reduction in BAG1 in response to H_2_O_2_; however, this was followed by a significant and time-dependent increase. **G** The H_2_O_2_-induced upregulation of BAG1 was replicated in the frontal cortex of both HIV animal models, with each displaying significantly increased levels of BAG1 RNA. **p < 0.01; ***p < 0.001. Scale bar equals 20 μM; ×60 magnification.

Given their complimentary roles in autophagy (i.e., BAG3) and proteasomal degradation (i.e., BAG1), we next determined whether a downregulation in BAG3 was associated with a compensatory upregulation of BAG1^51,52^. In primary neurons expressing either Tat or Nef, no significant variation in BAG1 protein was observed, either by immunoblot or fluorescent microscopy (Fig. 3A, C). Variation in BAG1 protein reflected its predominant rat neuronal isoforms, L and M, consistent with previous observations in BAG1 protein levels and calculated molecular weights^53^. However, significant reductions in BAG1 RNA were found in response to either viral protein (Fig. 3B). Differences in BAG1 RNA reflected both its predominant rat neuronal isoforms^54^.

In contrast, cells exposed to H_2_O_2_ displayed significantly elevated levels of BAG1. Immunocytochemistry indicated increases in BAG1 protein levels following 6 h of H_2_O_2_ treatment, while immunoblot results indicated this increase was dependent on the duration of treatment (Fig. 3C, D; Supplementary Fig. 4A). Moderate H_2_O_2_ dosages (100 μM) and minimal dosages (50 μM) were unsuccessful in perturbing BAG1 protein levels (Supplementary Fig. 4B). Taken together, these results are consistent with the time-dependent decreases in BAG3 found in previous experiments and suggest a decrease in BAG3 may precede the increase in BAG1 levels. However, unlike the additive effect on BAG3, the combination of H_2_O_2_ with Tat or Nef did not exacerbate the increase in BAG1 (Fig. 3E; Supplementary Fig. 4C).

Consistent with the downregulation induced by HIV-1 viral proteins (i.e., Fig. 3B), neurons exposed to H_2_O_2_ initially displayed significant decreases in BAG1 RNA, most notably following 2 h of treatment; however, H_2_O_2_ also triggered a significant increase in BAG1 RNA after this initial decrease, although levels did not completely return to baseline (Fig. 3F). Such an inverse regulation of BAG1, compared to BAG3, was also observed in each animal model, with iTat and Tg26-HIV mice displaying significantly increased measures of BAG1 RNA compared to controls (Fig. 3G).

### H_2_O_2_-sensitive proteins and antioxidant effects on BAG3

**Fig. 4:**
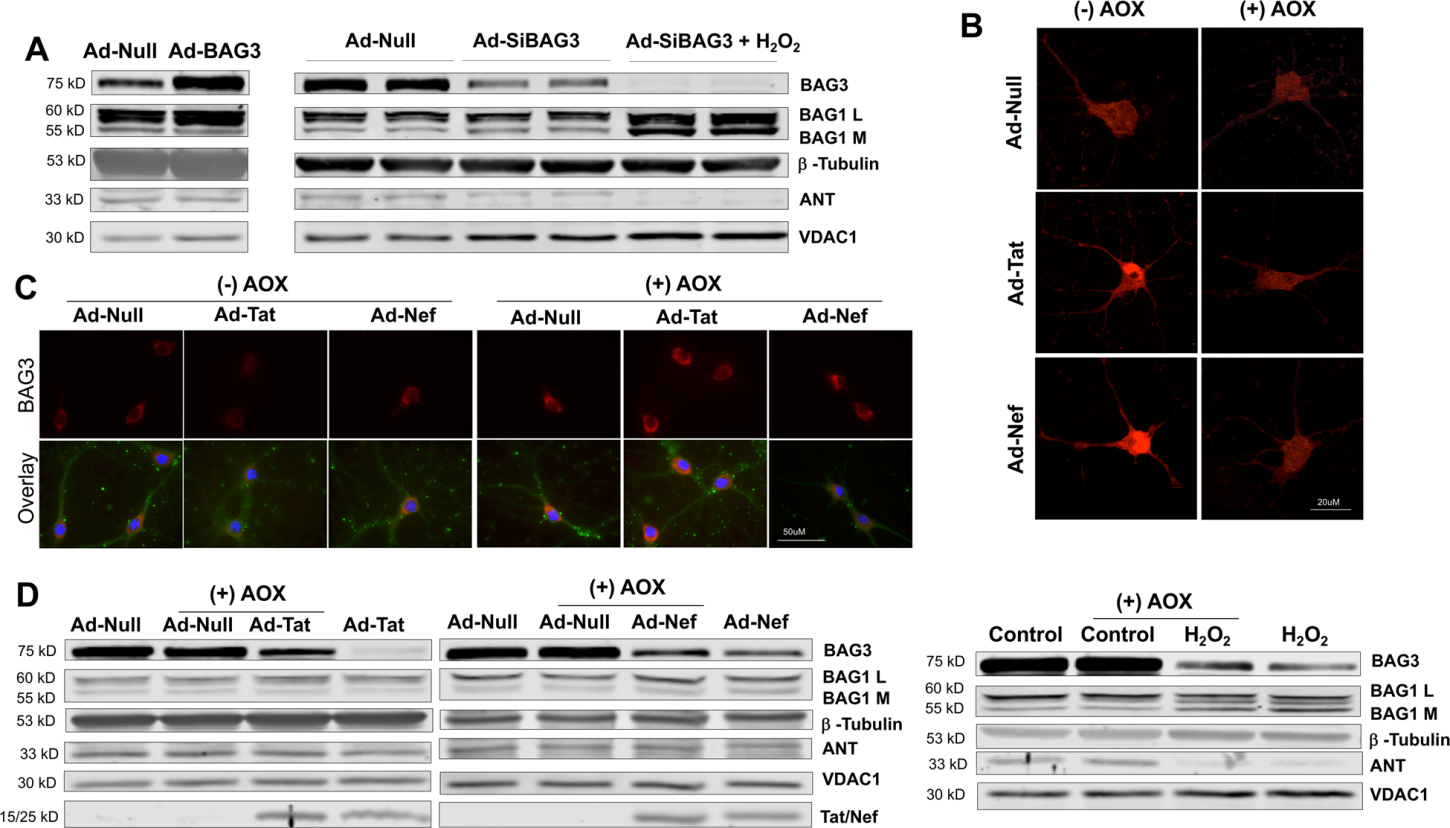
H_2_O_2_-sensitive proteins and antioxidant effects on BAG3. **A** Immunoblotting results indicated H_2_O_2_ treatment following BAG3 knockdown in neurons resulted in increased BAG1 protein, but alterations in BAG1 were not observed following the overexpression or knockdown of BAG3 alone; moreover, decreases in BAG3 were correlated with increased dysregulation of proteins responsible for maintaining mitochondrial membrane permeability, VDAC1, and ANT. **B** Live-cell microscopy indicated antioxidant treatment inhibited the increase in ROS otherwise induced by viral protein expression. **C** Immunocytochemistry suggested a decrease in neuronal oxidative stress was capable of partially preserving BAG3 protein levels. **D** Compared to control conditions, antioxidant treatment was not capable of fully preserving BAG3 levels, nor was it capable of inhibiting the dysregulation of mitochondrial proteins. Scale bar equals 20 μM; ×60 magnification. Scale bar equals 50 μM; ×100 magnification.

Additional data indicated the dynamic effects on neuronal BAG1 and BAG3 due to H_2_O_2_ was associated with alterations in several mitochondrial proteins as well as polypeptides known to interact with BAG family members. Primary neurons treated with H_2_O_2_ following BAG3 knockdown displayed a substantial BAG1 upregulation, while significant alterations in BAG1 were not observed following the overexpression or knockdown of BAG3 alone (Fig. 4A; Supplementary Fig. 5A). These decreases in BAG3 were associated with increased dysregulation of proteins responsible for maintaining mitochondrial membrane permeability; here, decreases in ANT and increases in VDAC1 following BAG3 knockdown were exacerbated by H_2_O_2_ (Fig. 4A; Supplementary Fig. 5B). In addition, neurons exposed to H_2_O_2_ maintained alterations in several other proteins known to bind with and influence BAG family member functioning, including Bcl-2, LC3, and Hsc/Hsp 70 (Supplementary Fig. 5C).

The direct inhibition of oxidative stress in neurons was capable of partially preserving BAG3 levels. Initial experiments employing live-cell microscopy confirmed the application of antioxidants (AOX) was capable of inhibiting the increase in ROS otherwise induced by viral protein expression (Fig. 4B; Supplementary Fig. 5D). Due to cross-reactivity between MitoSOX reagents with H_2_O_2_ in solution, we were unable to reliably discern if direct application of H_2_O_2_ was ameliorated by AOX. While immunoblotting and fluorescent microscopy confirmed AOX partially preserved BAG3 protein levels otherwise reduced by HIV-1 viral proteins, results suggested AOX treatment was not capable of mitigating the dysregulation induced by H_2_O_2_ (i.e., alterations in BAG3, BAG1, and mitochondrial proteins) (Fig. 4C, D; Supplementary Fig. 6A–C).

### Neuronal electrophysiology

**Fig. 5:**
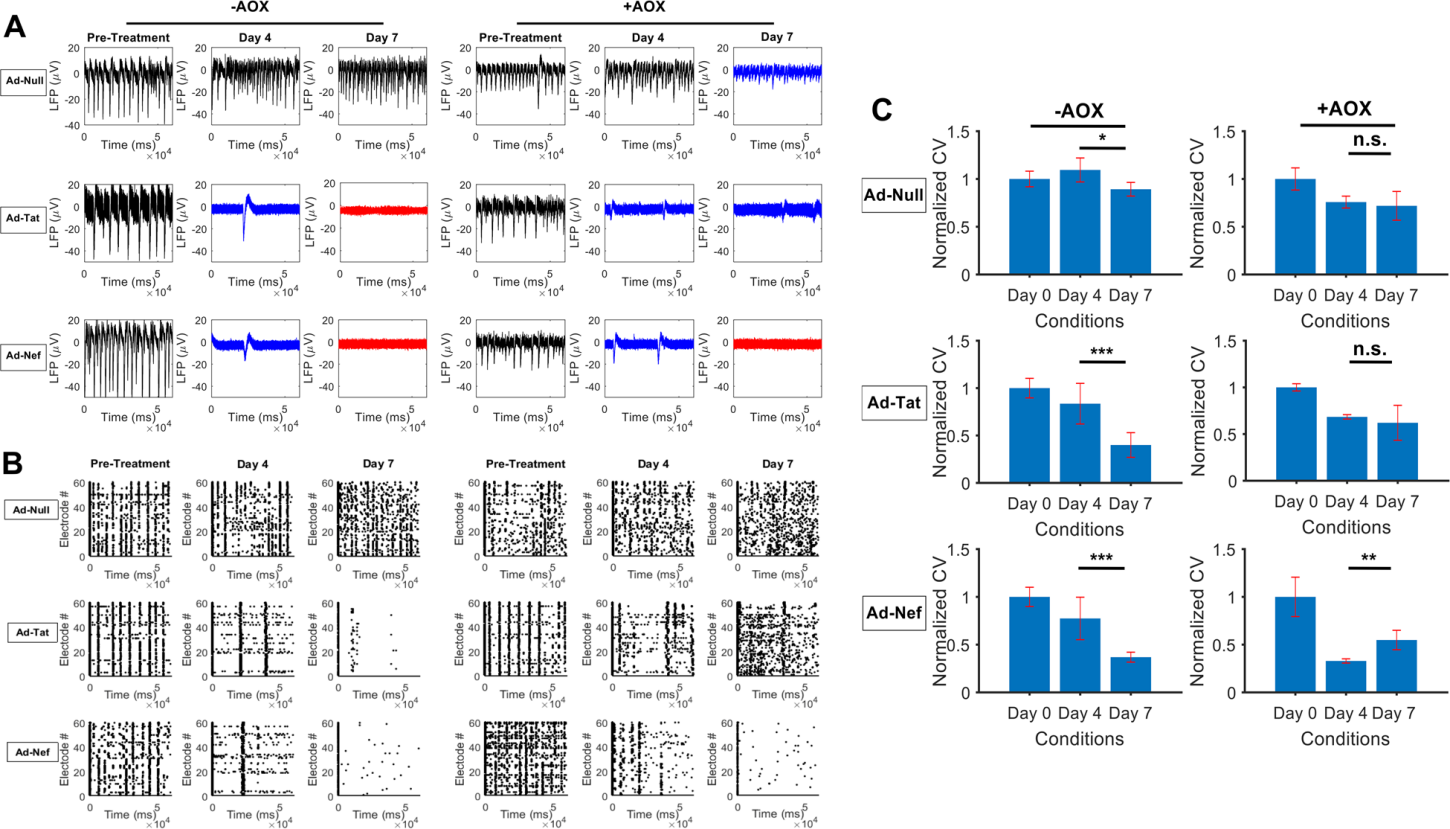
Neuronal Electrophysiology. **A** Local field potentials (LFPs) from MEA recordings indicate the expression of Tat and Nef significantly suppressed neuronal activity, where black, blue, and red color indicate normal, suppressed, and silenced activities, respectively. AOX treatment of Tat expressing neurons was capable of inhibiting further attenuation in network firing frequencies and partially stabilizing amplitudes, while treatment in Nef expressing neurons only partially preserved firing amplitudes under acute conditions. **B** Raster plots demonstrated the highly synchronous and regular firing activity at baseline was fully mitigated by the expression of Tat or Nef. AOX treatment resulted in a partial restoration of synchronous activity in the Tat expressing group, but not Nef. **C** Analyses of correlation-based conduction velocities revealed the application of AOX prevented further attenuation in temporal firing dynamics in Tat expressing neurons, and improved activity in the Nef expressing group. *p < 0.05; **p < 0.01; ***p < 0.001.

Assessment of electrophysiological measurements indicated AOX was capable of partially preserving neuronal functioning otherwise dysregulated by HIV-1 viral proteins, particularly Tat (Fig. 5A; Supplementary Fig. 7A). As shown by LFP signals and quantifications, Tat and Nef suppressed neuronal activity in terms of amplitude and spiking frequency compared with Ad-Null treated controls. This behavior continued to develop leading to complete attenuation of neuronal activity under Tat and Nef expression. Surprisingly, AOX treatment of Tat expressing neurons was capable of preserving activity measures by inhibiting further attenuation in network firing frequencies and partially stabilizing amplitudes. While AOX treatment of Nef expressing neurons partially preserved firing amplitudes under acute conditions, it was incapable of inhibiting long-term attenuation in amplitudes or preserving firing frequencies at any time point.

In evaluating synchronous and spontaneous activity of neuronal populations, analysis of baseline measurements illustrated regular bursting activity in all MEAs (Fig. 5B). In the absence of AOX treatment, neurons transduced with Ad-Tat or Ad-Nef consistently displayed disrupted firing patterns across electrodes. Treating neurons with AOX resulted in a partial restoration of synchronous activity in the Tat expressing group, but not Nef. Analyses of conduction velocities quantified the speed at which similar patterns of activity propagated across MEAs, whether in the form of spikes or subthreshold oscillations (Fig. 5C). In agreement with the measures above, data indicated suppressed conduction velocities in neuronal cultures expressing Tat or Nef compared to negative controls. Interestingly, the application of AOX prevented further attenuation in activity patterns amongst Tat expressing neurons, and improved activity in the Nef expressing group. Further analyses of frequency content as well as synchronous activity within clusters of neurons indicated minimal benefits upon AOX treatment, and confirmed the adverse effects of Tat and Nef (Supplementary Fig. 7B, C). Notably, the effects of AOX under H_2_O_2_ conditions could not be reliably examined due to peroxide-induced electrical interference during recording sessions.

## Discussion

The current findings address the potential for HIV-1 viral proteins and H_2_O_2_ to induce variation in neuronal BAG family members, a family of proteins responsible for efficient PQC. Both Tat and Nef viral proteins facilitated elevations in intraneuronal ROS and decreased BAG3 levels, while also disrupting neuronal firing activity. H_2_O_2_ exacerbated the reduction in BAG3 and induced an upregulation of BAG1. Such inverse ratios were maintained in two animal models which express viral proteins in the CNS, the doxycycline-inducible Tat (iTat) and Tg26 mouse. Interestingly, the inhibition of oxidative stress in primary neurons was capable of partially preserving electrophysiological functioning and BAG3 levels otherwise altered by HIV-1 viral proteins. Results support the role of H_2_O_2_ as a modulator of neuronal PQC by regulating BAG family members in response to HIV-1 viral proteins or other potential sources of oxidative stress, such as aging (Supplementary Fig. 8).

The pattern of BAG3 and BAG1 regulation reported herein warrants consideration and emphasizes the implications of cell-specific experimental paradigms. As oxidative stress and aging can mitigate proteasome functioning while also increasing concentrations of aggregate-prone polypeptides, a compensatory yet disproportionate demand for BAG3 over BAG1 has been hypothesized in response to perturbation in neuronal homeostasis^44,55^. Indeed, in a cell line model of aging as well as neural cell lines clonally selected for resistance to oxidative stress, a group of researchers recently reported increasing BAG3 and decreasing BAG1 in response to H_2_O_2_^42,43,45^. This group reported similar results in aged brain samples containing both neurons and glia (i.e., tissue homogenates, ex vivo hippocampal slices)^42^. However, researchers indicated such BAG regulation was specific to neurons (see Fig. 2F in Gamerdinger et al., 2009), consistent with results obtained here (see Supplementary Fig. 3D). Therefore, the direction of BAG3 and BAG1 regulation by H_2_O_2_ in the current experiments may be reflective of cell-specific responses obtained from primary neurons, whereas prior experimental procedures may reflect general cellular adaptations in the neural milieu. In light of accumulating evidence distinguishing the molecular biology of neurons from other cell types in the brain, particularly in response to aging and neuropathogenesis, such cell-specific responses are plausible and increasingly relevant^56,57^.

Current experiments provided further mechanistic insights for the biomolecular underpinnings of HIV neuropathogenesis, particularly regarding the consequences of viral proteins on homeostatic functioning in neurons^21,58^. Further investigations are necessary to validate the physiological relevance of these data in the context of recombinant Tat and Nef proteins. In addition, while we reported increased BAG1 and decreased BAG3 RNA in brain tissues of two different HIV animal models, this pattern of regulation may reflect a compensatory adaptation between neuronal and non-neuronal cells to confer neuroprotection during adulthood^59,60^. Therefore, future studies should determine if this regulation of neuronal BAG proteins in animal models of HIV is age-dependent, given that cell-specific population densities and functioning can vary in the aging CNS, compared to adolescence or adulthood^61^.

Taken together, our results suggest the regulation of oxidative stress in conjunction with PQC modulators may result in novel therapeutic opportunities in HAND. Specifically, data from HIV-1 proteins indicated antioxidants could partially preserve neuronal electrophysiology and BAG3 levels. This is consistent with the otherwise adverse consequences of Tat and Nef, which include the generation of abnormal ROS and impairments in PQC^17,29,62,63^. While the reliability of antioxidant therapeutics remains debated, some reports suggest their application in combination with other treatments can result in enhanced outcomes for neurodegenerative conditions, including AD^64,65^. As the selective augmentation of BAG proteins has proved efficacious in the context of other age-related neurological diseases, targeted PQC therapies in combination with antioxidant therapies may provide novel treatments amongst our aging HIV population^66–69^.

## Materials and methods

### Primary neuronal culture and cell treatment

Tissue preparation and cell cultures were performed according to Temple University’s Institutional Animal Care and Use Committee and the National Institute of Health (NIH) guidelines. As described previously, primary neuronal cultures were prepared using dissected E18 prenatal rat embryonic brains^46,70^. After digestion in trypsin solution (0.25%), neurons were plated on tissue culture plates, slides, or microelectrode arrays, MEAs (see below), that were coated with poly-D-lysine (Sigma-Aldrich, St. Louis, MO) as well as laminin (Invitrogen 23017, USA). Cells were maintained 17 days (25 days for MEA studies) in vitro (DIV) prior to treatments, at which point cells were randomly assigned to conditions and experiments were initiated. All experiments were completed in triplicate to confirm the reliability of results and to obtain appropriate power for statistical analyses.

To ensure no degradation of reagents, H_2_O_2_ treatments proceeded using indicated dilutions prepared from stock immediately prior to their application (VWR BDH7690, USA); solutions were prepared under sterile conditions with diH_2_O (100 μL total volume). Neurons were transduced with Ad-Null (Vector Biolabs, Malvern, PA), Ad-Tat (made in-house), Ad-Nef (made in-house), Ad-siBAG3 (Vector Biolabs, Malvern, PA), and Ad-BAG3 (Vector Biolabs, Malvern, PA). To produce viral constructs in-house for transduction, cDNA was cloned from HIV strain 89.6 into compatible restriction sites of the shuttle plasmid pDC515(IO) and subsequently rescued by co-transfection with pBHGfrtDeltaE1,E3FLP in 293 IQ cells (Microbix Corporation, Mississauga, Ontario, Canada). The Ad was then plaque purified, amplified, and subsequently purified on cesium chloride (CsCl) gradient centrifugation. Plaque purified virus was dialyzed against elevated salt and MgCl_2_ for further purification, provided that CsCl does not generate entirely pure preparations. Collected viral particles were diluted for concentration measurement at OD260, while the virus concentration was calculated based on number of particles/ml. MOI was maintained at 1 for all vectors throughout procedures. Transduction durations were maintained in accordance with previous experiments in our laboratory (72 h). To assess the combined effect of H_2_O_2_ with viral proteins, H_2_O_2_ treatments were initiated after the transduction period; this time point remained consistent for all H_2_O_2_ experiments. Antioxidant (AOX) treatments were applied in a manner previously described^71,72^; here, a proprietary antioxidant solution at recommended dilutions (1:1000 in culture media; 100 μL total volume) was applied in culture for 24 h following transduction (A1345; Sigma-Aldrich, St. Louis, MO).

### Animal models

Animal procedures and protocols were performed according to Temple University’s Institutional Animal Care and Use Committee and the National Institute of Health (NIH) guidelines. Adult male mice (18–28 g) were singly housed in a temperature (21–23°) and humidity-controlled vivarium with constant airflow on a reverse 12-h light/dark cycle (lights on/off at 09:00 EST). Food and water were available ad libitum. Two mouse models previously in our laboratory were utilized in the present experiments. Tg26-HIV-transgenic mice are a widely used and well-described mouse model that encode the entire pNL4–3 HIV-1 genome, except a segment of gag/pol genes. This model expresses HIV viral proteins such as Tat and Nef, while exhibiting neuropathology observed upon HIV infection, including neurocognitive deficits. Doxycycline (DOX)-inducible GFAP promoter-driven HIV-1 Tat transgenic mice were provided by the Comprehensive NeuroAIDS Center. This model conditionally-expresses the HIV-1 Tat_1–86_ protein in an astrocyte-specific manner under the control of a GFAP-driven Tet-on promoter, which is activated in the presence of DOX. Both iTat and their control littermates were randomly selected to receive doxycycline hyclate (DOX) orally over the course of 6 weeks (S3888; Bioserv, Flemington, NJ). C57BL/6J mice maintained in our laboratory’s colony were utilized as controls. All mice in each group (*n* = 4) were euthanized at similar time points (~6 months old) and brain tissue was harvested for subsequent analyses. As many of the cognitive deficits manifested in HAND patients during the post-cART era are associated with aberrant functioning in cortical regions of the brain (e.g., attention, executive functions, learning), analyses focused on tissue from this area^73^.

### Metabolic and viability assays

To assess general metabolic activity, the MTT (3-(4,5-dimethylthiazol-2-yl)-2,5-dihhenyltetrazolium bromide) assay was used. Following treatment, primary rat neurons were incubated for 2 h at 37 °C in solution containing MTT according to the manufacturer’s recommendations (CT015; Sigma-Aldrich, St. Louis, MO). Measures from each sample were determined as the difference between 595 and 620 nm wavelength values obtained by spectrophotometry. To gauge neuronal cell death, the trypan blue exclusion procedure was used. Here, cell solutions were prepared by equal dilution with trypan blue dye (15250061; ThermoFisher, Waltham, MA) followed by quantification using disposable slides (C10228; ThermoFisher, Waltham, MA) and an automated cell counter (AMQAF1000; ThermoFisher, Waltham, MA).

### Western blotting

For immunoblotting experiments, neuronal and mouse brain samples (frontal cortex) were homogenized and lysed with RIPA buffer (25 mM Trizma base pH 7.6, 150 mM NaCl, 1% NP-40, 1% sodium deoxycholate, 0.1% SDS) containing a protease inhibitor cocktail (Sigma-Aldrich, St. Louis, MO). Protein concentrations were assessed by means of a standardized Bradford assay (Bio-Rad, Hercules, CA). SDS-polyacrylamide gels, 10–12%, and nitrocellulose membranes (LI-COR, Inc., Lincoln, NE) were used for electrophoretic protein separation and transfer, respectively. To reduce non-specific binding and background, membranes were blocked in Odyssey (LI-COR) blocking buffer (1 h, room temperature). Primary (1:1000, overnight, 4 °C) and secondary (1:10,000, 1 h, room temperature) antibodies were applied followed by imaging on the Odyssey CLx Imaging System (LI-COR). Protein levels were assessed and standardized to appropriate loading controls by means of optical density analysis using Image Studio acquisition software. The following primary antibodies were used: ANT (Santa Cruz; SC11433), β-Tubulin (Sigma; T8578), Bcl-2 (Sant Cruz; SC7382), BAG1 (Santa Cruz; SC939), BAG2 (Novus Biologicals; NBP159086), BAG3 (Proteintech; 105991AP), BAG5 (Proteintech; 266281AP), BAG 6 (R&D Systems; AF6438), BAX (Santa Cruz; SC493), GAPDH (Santa Cruz; SC32233), Hsc/Hsp 70 (Sant Cruz; SC24), LC3 (Santa Curz; L8918), Nef (Abcam; ab42355), Tat (NIH Aids Reagent Program; R705), VDAC1 (Santa Cruz; SC8017).

### Immunocytochemistry

Following fixation (4% paraformaldehyde), blocking (1% BSA), and washing, neurons were labeled with the following primary antibodies (1:100, overnight, 4 °C): β Tubulin (Sigma; T8578), BAG1 (Santa Cruz; SC376848), BAG3 (Proteintech; 105991AP) MAP2 (Cell Signaling; 4542). Alexa Fluor®secondary antibodies (1:500) (ThermoFisher, Waltham, MA) and VectaShield with DAPI medium (Vector Laboratories, Burlingame, CA) were used for labeling and mounting, respectively. Imaging utilized a fluorescence microscope (BZ-X710; Keyence, Osaka, Osaka, Japan) and image processing employed the Hybrid Cell Count module within the BZ Image Analyzer software (Keyence) to obtain optical density measurements expressed in μM per cell.

### ROS quantification

To assess levels in reactive oxygen species (ROS), cells were incubated with the mitochondrial oxygen free radical indicator MitoSOX-red (Life Technologies, Carlsbad, CA) for 30 min at 37 °C. Slides were then mounted for confocal imaging in an open perfusion microincubator (PDMI-2; Harvard Apparatus) and images were obtained at 561 nm excitation by using a confocal microscope (810; Carl Zeiss, Oberkochen, Germany). Optical densitometry quantifications were expressed as fluorescence intensity normalized to areas as previously described^74^.

### RNA isolation and cDNA preparation

Total RNA for each neuronal and mouse brain sample (frontal cortex) was processed using the Trizol (ThermoFisher) extraction protocol followed by RNA purification using Direct-zol™ RNA MiniPrep Plus (Zymo Research, Irvine, CA) in accordance with manufacturer specifications. cDNA synthesis on total RNA samples utilized with the High Capacity cDNA Reverse Transcription Kit (Thermo Fisher Scientific).

### Real time-quantitative RT-PCR (qRT-PCR)

Sequence design for forward and reverse primers utilized the most to date reference genomes followed by submission to NCBI’s Basic Local Alignment Search Tool (BLAST) to ensure the selected primers do not display similarity to other sequences. The sensitivity and specificity of primers were checked by RT-PCR using FailSafe PCR Kit (Lucigen, Middleton, WI). All qPCR reactions were conducted with the LightCycler96^®^ (Roche) using the SYBR™ Green master mix (Applied Biosystems, ThermoFisher), according to the manufacturer’s specifications. Relative quantity was normalized to Actin expression. Primer sequences were as follows: Actin (Forward-5′-CAGGTCCAGACGCAGGATGGC-3′; Reverse-5′-CTACAATGAGCTGCGTGTGGC-3′), BAG1 (Forward-5′-AGATGGTCCAGACGGAGGAA-3′; Reverse-5′-CAGATACCTC CAAGTCCTTCAGC-3′), BAG3 (Forward-5′-GGCCCTAAGGAAACTGCAT-3′; Reverse-5′-GGGAATGGGAATGTAACCTG-3′), Tat (Forward-5′-GGAATTCACCATGGAGCC AGTA-3′; Reverse-5′-CGGGATCCCTATTCCTTCGGGC-3′).

### Microelectrode array (MEA)

Neuronal activities were recorded pre-treatment (Day 0) and 96 h (Day 4) as well as 168 h (Day 7) after transduction. To ensure the assessment of AOX effects was temporally consistent with AOX effects observed in other experiments, recordings were conducted 24 h after AOX was added to half of the cultures post-transduction (i.e., 96 h). Media with or without AOX was then maintained until the final recording session on Day 7 to assess potential chronic effects of AOX treatment on neuronal functioning. In each recording session, multiple minutes of recordings were performed to acquire local field potential (LFP) of neuronal extracellular action potential at 2000 Hz (2 kHz) from 60 electrodes simultaneously. Recorded data were then transferred as numerical values in microvolts to MATLAB (Mathworks, Natick, MA) for pre- and post-processing. Low-pass filtering with cut-off frequency *f*_c_ = 300 Hz was applied to all recording data prior to data analysis.

LFPs of different experimental groups were visualized in MATLAB with representative channels in a 60,000 ms course (120,000 sampled points for each signal). To depict the network-wide activation and provide a measure of activity beyond a single electrode, raster plots were generated based on the time at which a spike was detected (activity exceeding three times standard deviation of the raw data) for all channels, and visualized for all time points as well as experimental groups. To show the rate at which neuronal activation travels from the source across populations, the cross-correlation values between pairs of electrodes and their distance were used to compute conduction velocities (cm/s). These data were then normalized (nondominsionalized) with respect to initial conditions to reflect the treatment effects on wave propagation rates. For the technical details refer to ref. ^70^. To examine frequency content of neuronal oscillations, time domains LFPs of all electrodes were transformed and analyzed to frequency domains using fast Fourier transform (FFT). The resulting mean and standard deviations were plotted and analyzed for each condition. To determine the effect of different experimental conditions on network-wide synchronous clusters of neurons, rank correlation matrices were first calculated across electrodes. Subsequently, hierarchical clustering identified clusters of electrodes that exhibited similar or comparable correlation patterns, number of clusters and the magnitude of correlation, which were presented in a heat map format.

### Statistical analyses

Statistical procedures were optimized based on the experimental designs as well as the consistent, continuous nature of the measured variables across assays. Independent samples *t*-tests were primarily employed to compare measures between control and experimental conditions across assays. For those assays which required multiple comparisons either between or within samples, analysis of variance (ANOVA) followed by appropriate post hoc comparisons and corrections were conducted. All data were screened for normality. For MEA quantifications, mean and standard deviations of the number of detected spikes were computed across 60 electrodes for each time point and experimental group. Mean and standard deviations of maximum LFP amplitudes were also obtained for each time point/experimental group. All statistical analyses were conducted utilizing SPSS v 22 (IBM, Armonk, NY) or MATLAB (Mathworks).

## Supplementary information

Supplementary Figure Legends

S1

S2

S3

S4

S5

S6

S7

S8
