# Supplementary material for: Neuromodulation of BAG co-chaperones by HIV-1 viral proteins and H2O2: implications for HIV-associated neurological disorders
Source: Cell Death Discov. 2021 Mar 26;7:60. doi: 10.1038/s41420-021-00424-0 (PMC7997901; doi:10.1038/s41420-021-00424-0)
Supplement: Supplementary file 1 — Supplementary Figure Legends [file 41420_2021_424_MOESM1_ESM.docx]

**Supplementary Figures**

**S1. A)** Immunocytochemistry demonstrated HIV-1 viral protein expression (Tat or Nef) decreased levels of BAG3. **B)** Quantification of immunocytochemistry and immunoblotting confirmed significant reductions in BAG3 following expression of Tat or Nef. **C)** Additional live-cell microscopy images and quantifications depicted the elevation in neuronal ROS following viral protein expression. **D)** Quantifications of western blots from HIV-1 animal models illustrated a significant decrease in levels of BAG3 protein. **E)** qPCR results validating expression of Tat. * *p* < .05; ** *p* < .01; *** *p* < .001. Scale bar equals 50uM; 20x magnification. Scale bar equals 20uM; 60x magnification.

**S2. A)** MTT assays demonstrated significant reductions in neuronal metabolic activity following 6 Hr of H_2_O_2_ treatment at dosages greater than 150uM. **B)** Trypan blue staining suggested 6 Hr of H_2_O_2_ treatment at dosages greater than 250uM results in significantly increased percentages of non-viable neurons. **C)** Time course experiments utilizing MTT assays indicated 250uM H_2_O_2_ treatment significantly impaired metabolic activity in neurons between 1-6 Hrs, while further metabolic reductions were not observed after 8 Hr. **D)** Using trypan blue staining, time course experiments also indicated 250uM H_2_O_2_ significantly decreased neuronal viability after 8 Hrs of treatment. ** *p* < .01; *** *p* < .001.

**S3. A)** Immunocytochemistry, as well as quantifications, confirmed significantly attenuated levels of neuronal BAG3 in response to H_2_O_2_. **B)** Western blotting demonstrated neurons treated with moderate dosages (100uM) for extended durations (8 hours) emulated significant declines in BAG 3 protein, but minimal dosages (50uM) were unsuccessful in perturbing BAG3 regardless of treatment duration. **C)** Immunoblotting illustrated the co-expression both Tat and Nef proteins did not result in an additive effect on neuronal BAG3 reduction. **D)** As indicated by western blot, decreases in BAG3 due to H_2_O_2_ appeared to be neuronal-specific, provided that primary rat astrocytes failed to show similar sensitivity to H_2_O_2_ treatment. *** *p* < .001. Scale bar equals 50uM; 20x magnification.

**S4. A)** Immunocytochemistry, as well as quantifications, confirmed increases in BAG1 protein levels following 6 hours of H_2_O_2_ treatment. **B)** Moderate (100uM) or minimal H_2_O_2_ dosages (50uM) were unsuccessful in perturbing BAG1 protein levels regardless of treatment duration. **C)** Quantifications of immunoblots illustrated the combination of H_2_O_2_ with either Tat or Nef did not significantly exacerbate the H_2_O_2_-induced increase in BAG1. ** *p* < .01. Scale bar equals 50uM; 20x magnification.

**S5. A)** Quantification of immunoblot results confirmed significant alterations in BAG1 protein levels in neurons treated with H_2_O_2_ following BAG3 knockdown, not following the overexpression or knockdown of BAG3 alone. **B)** Quantification of immunoblot results indicated decreases in ANT and increases in VDAC1 following BAG3 knockdown were exacerbated by H_2_O_2_. **C)** Additional western blots suggested neurons exposed to H_2_O_2_ treatments also maintained alterations in several proteins known to interact with BAG family members, including Bcl-2, LC3, and Hsc/Hsp 70. **D)** Live-cell microscopy quantifications confirmed the application of AOX was capable of inhibiting the increase in ROS otherwise induced by viral protein expression. * *p* < .05, ** *p* < .01, *** *p* < .001

**S6. A)** Immunocytochemistry results demonstrated antioxidant treatment in neurons was capable of partially protecting BAG3 levels in response to Tat or Nef. **B)** Quantification of immunoblot results confirmed the significant decreases in BAG3 induced by HIV-1 viral proteins was inhibited by antioxidant treatment. **C)** Quantification of immunocytochemistry results confirmed the significant decreases in BAG3 induced by HIV-1 viral proteins was inhibited by antioxidant treatment * *p* < .05, ** *p* < .01. Scale bar equals 50uM; 20x magnification.

**S7. A)** Local field potentials (LFPs) from MEA recordings suggest that Tat and Nef expressing neurons displayed a gradual drop in firing activity post-transduction. AOX treatment inhibited further suppression in firing frequency or amplitude otherwise caused by Tat and only partially preserved firing amplitudes under acute conditions in Nef expressing neurons. **B)** Clusters of synchronously firing neuronal sub-populations were desynchronized by expression of either viral protein, and AOX application was unable to restore such activity within distinct populations of neurons. The heat color heat maps provide information on the magnitude and direction of correlation, with bright red cells showing positive and bright green cells denoting negative correlation among the electrodes, while darker colors indicate the lack of strong correlation. **C)** Low frequency oscillations of neuronal activity analyzed using fast Fourier transform (FFT) confirmed Tat and Nef expression led to complete attenuation of neuronal oscillations smaller than 5 Hz compared to proportional frequencies in control conditions. Although Tat-expressing neurons treated with AOX displayed minor oscillatory resiliency, no beneficial effects were observed in Nef conditions. *** *p* < .001.

**S8)** Findings revealed the HIV proteins Tat and Nef induced elevations in intraneuronal ROS, decreased BAG3 levels and disrupted neuronal firing activity. As a potent ROS, H_2_O_2_ exacerbated this reduction in BAG3 and induced an upregulation of BAG1, while also dysregulating proteins responsible for maintaining mitochondrial membrane permeability, VDAC1 and ANT. The inhibition of oxidative stress in primary neurons was capable of partially preserving electrophysiological functioning and BAG3 levels otherwise altered by HIV-1 viral proteins, indicting the causal role of ROS in mediating of neuronal homeostasis. Taken together, results support the role of H_2_O_2_ as a modulator of neuronal PQC by regulating BAG family members in response to HIV-1 viral proteins or other potential sources of oxidative stress, such as aging.
