# Supplementary figures and images for: Neuromodulation of BAG co-chaperones by HIV-1 viral proteins and H2O2: implications for HIV-associated neurological disorders
Source: Cell Death Discov. 2021 Mar 26;7:60. doi: 10.1038/s41420-021-00424-0 (PMC7997901; doi:10.1038/s41420-021-00424-0)

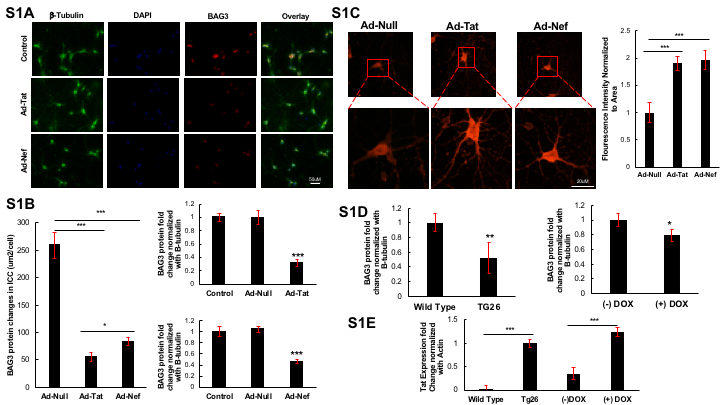

Supplement: Supplementary file 2 — S1 [file 41420_2021_424_MOESM2_ESM.png]

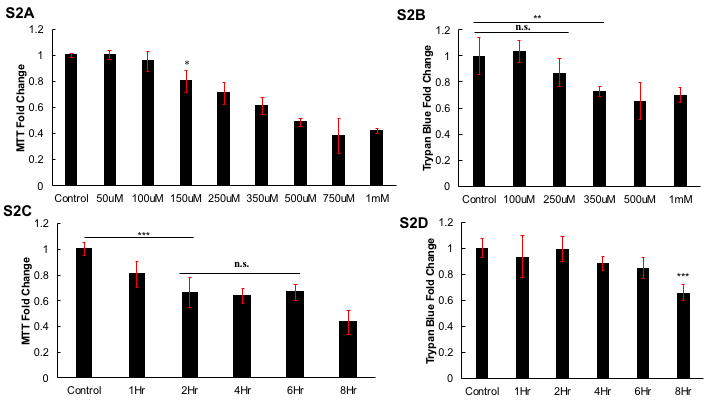

Supplement: Supplementary file 3 — S2 [file 41420_2021_424_MOESM3_ESM.png]

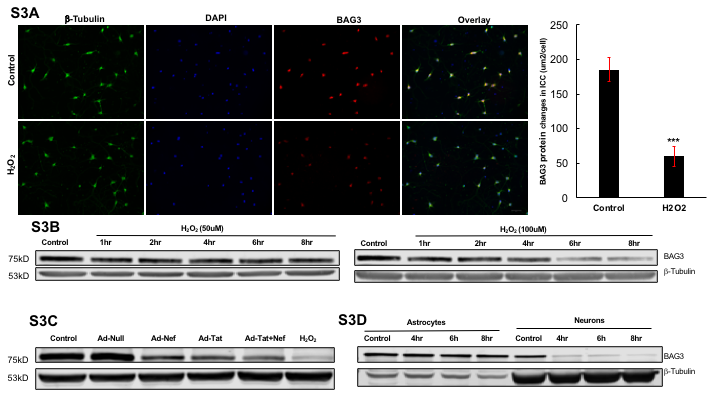

Supplement: Supplementary file 4 — S3 [file 41420_2021_424_MOESM4_ESM.png]

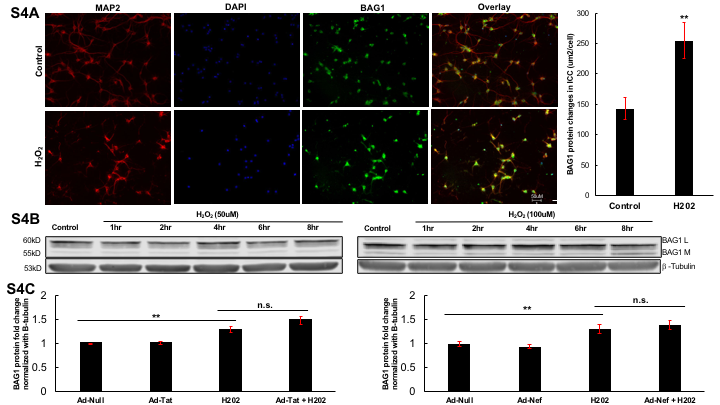

Supplement: Supplementary file 5 — S4 [file 41420_2021_424_MOESM5_ESM.png]

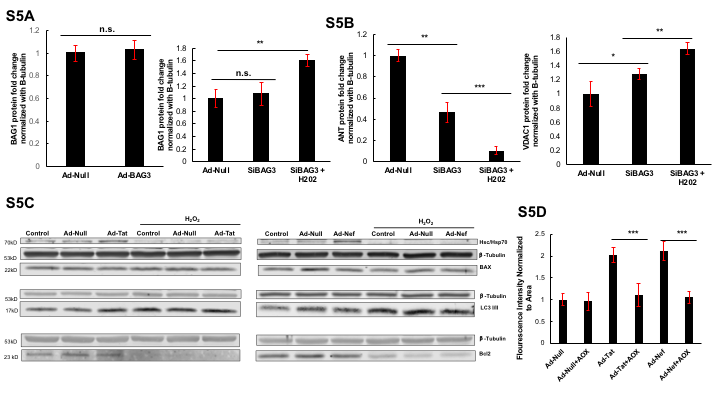

Supplement: Supplementary file 6 — S5 [file 41420_2021_424_MOESM6_ESM.png]

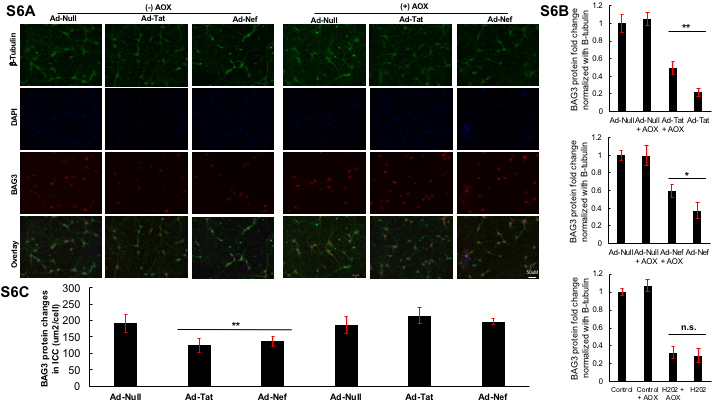

Supplement: Supplementary file 7 — S6 [file 41420_2021_424_MOESM7_ESM.png]

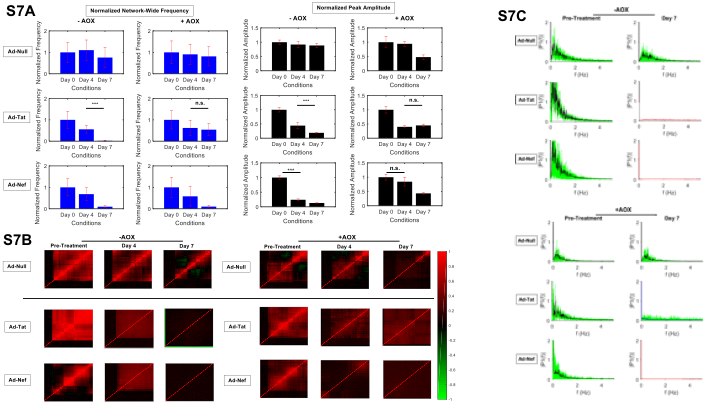

Supplement: Supplementary file 8 — S7 [file 41420_2021_424_MOESM8_ESM.png]

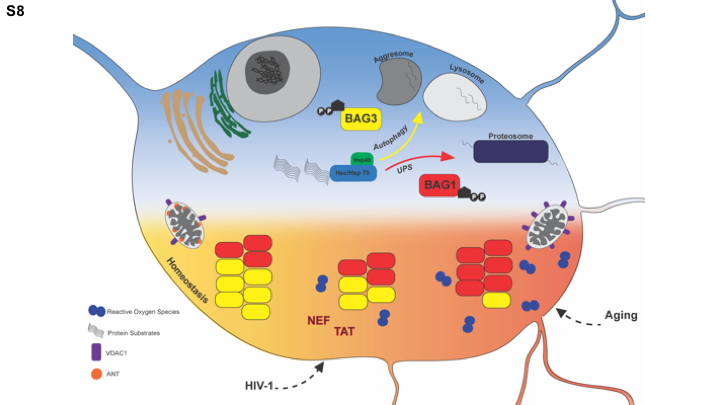

Supplement: Supplementary file 9 — S8 [file 41420_2021_424_MOESM9_ESM.png]
